# AI in Root Canal Morphology: A Detailed Bibliometric Analysis of Research Trends and Global Contributions

**DOI:** 10.1002/cre2.70269

**Published:** 2025-12-29

**Authors:** Waseem Hassan, Vini Mehta, Niher Tabassum Singdha, Mohmed Isaqali Karobari

**Affiliations:** ^1^ Institute of Chemical Sciences University of Peshawar Peshawar Khyber Pakhtunkhwa Pakistan; ^2^ Department of Dental Research Cell Dr. D. Y. Patil Dental College and Hospital, Dr. D. Y. Patil Vidyapeeth Pune Maharashtra India; ^3^ Department of Dental Research, Saveetha Medical College and Hospitals, Saveetha Institute of Medical and Technical Sciences Saveetha University Chennai Tamil Nadu India; ^4^ Department of Conservative Dentistry and Endodontics, Saveetha Dental College and Hospitals, Saveetha Institute of Medical and Technical Sciences Saveetha University Chennai Tamil Nadu India; ^5^ Department of Restorative Dentistry & Endodontics, Faculty of Dentistry University of Puthisastra Phnom Penh Cambodia

**Keywords:** artificial intelligence, bibliometric analysis, co‐authorship network, root canal morphology, thematic analysis

## Abstract

**Background:**

Artificial Intelligence (AI) has emerged as a transformative tool in various scientific disciplines.

**Objective:**

The study aims to provide a comprehensive bibliometric analysis of the literature on AI and root canal morphology, examining publication trends, author contributions, and thematic focus areas.

**Methods:**

Data were retrieved from the Scopus database using the search string “Artificial Intelligence” OR “AI” AND “Root” OR “Root canal morphology,” covering publications from 1997 to 2024. Only articles and reviews were included. Vosviewer software was employed to visualize co‐authorship networks and perform co‐word analysis.

**Results:**

The analysis encompassed 81 papers, 433 authors, and 274 departmental contributions across 31 countries. The documents were published in 52 journals. The analysis revealed a global collaboration network with significant contributions from diverse institutions and countries. By co‐word analysis, the focus of the 81 papers is categorized into 25 distinct research areas. The thematic analysis identified key research areas and emerging trends within the literature. The findings highlight AI applications' increasing interest and interdisciplinary nature in root canal morphology research.

**Conclusion:**

This study provides a detailed overview of the research landscape concerning AI and root canal morphology. It identified leading contributors and institutions and presented a structured view of research themes.

## 1 Introduction

Root canal morphology is the study and understanding of a tooth's internal anatomy, encompassing the shape, size, number, and arrangement of the canals within the tooth roots. Each tooth has a distinct canal structure, which can differ significantly from person to person and within the same mouth (Ahmed et al. 2017). The complexity of this morphology is frequently characterized by differences in canal numbers, curvatures, and the existence of lateral canals, bifurcations, and other anatomical anomalies (Ahmed et al. 2017; Versiani et al. 2016). Accurate knowledge of this morphology is critical for effective root canal therapy (RCT), which removes infected or injured pulp tissue (Kazimierczak et al. 2024). A detailed understanding of the canal system enables dentists to efficiently clean and disinfect all parts of the root canal, reducing the danger of leaving contaminated tissue behind (Wolf et al. 2021), which can lead to recurrent infections, retreatment, and even tooth loss. The Vertucci classification has been widely used to describe root canal morphology since 1984. Sert et al. expanded it in 2004 to account for more complex canal systems, and Ahmed et al. (2017) refined it in 2017 with a single descriptive code representing canal morphology and root number. Traditional imaging modalities, such as periapical and bitewing radiographs and CBCT, have been used to assess root canal morphology (Taha et al. 2024). However, evaluation of these imaging techniques can be biased and vary based on surgeon's experience (Lai et al. 2023).

In the field of dentistry, artificial intelligence (AI) is developing quickly, especially in the interpretation of root canal morphology. AI tools like deep learning and machine learning have significantly improved endodontic treatment planning and diagnostic accuracy (Karobari et al. 2023). AI in endodontics involves classification, segmentation, clustering, regression, object detection, and landmark detection to enhance the analysis and interpretation of dental images. These tasks can enable precise diagnosis, treatment planning, and monitoring of root canal morphology, improving overall clinical outcomes. Additionally, image processing techniques like registration and enhancement are crucial for aligning, analyzing, and optimizing dental images for better accuracy in endodontic procedures (Ourang et al. 2024). AI models, like convolutional neural networks (CNNs), have shown improved accuracy in recognizing three‐dimensional root canal morphology (Wu et al. 2024) and vertical root fracture identification (Fukuda et al. 2020). Qiao et al. (2020) tested a neural network‐based multifrequency impedance method for root canal length measuring, which achieved approximately 95% accuracy, exceeding the classic dual‐frequency method. Furthermore, AI models have shown the ability to anticipate case difficulty (Qu et al. 2023), postoperative discomfort (Gao et al. 2021), and treatment prognosis (Bennasar et al. 2023), hence facilitating holistic treatment planning (Mohammad‐Rahimi et al. 2024). The growing volume of publications suggests that the application of AI in root canal morphology has progressed in theory and practice. Despite these promising improvements, current research on the use of AI in root canal morphology is limited in breadth due to a lack of research on alternative ML models such as Support Vector Machines or Neural Networks, a long‐term follow‐up on patients, and the need for research among bigger sample sizes (Wu et al. 2024; Bennasar et al. 2023).

A bibliometric analysis (Donthu et al. 2021; Hassan and Duarte 2024) of artificial intelligence applications in root canal morphology is required to fill these gaps. It will provide information on the contributions of various countries, institutions, journals, and scholars in the field of root canal morphology and will also systematically review the knowledge structure and map the global distribution of research activities in this field. Such a review will not only highlight the existing level of knowledge but will also indicate emerging research hotspots and areas that require additional inquiry, ultimately increasing the integration of AI in endodontic practice.

## 2 Materials and Methods

The data was retrieved from the Scopus database, which provides comprehensive coverage of scientific literature across various disciplines. The search string employed was: “Artificial Intelligence” OR “AI” AND “Root” OR “Root canal morphology.” The search was conducted with an open time frame to ensure a thorough exploration of the literature. We selected documents where the search string appeared in the “abstract,” ensuring the papers were directly relevant to our research focus. This approach maximizes the relevance of the included studies by focusing on abstracts where the key terms are explicitly discussed. Only those classified under the “Dentistry” category were included further to confirm the authenticity and relevance of the papers. This additional filter ensured that the selected documents were explicitly related to dental research, enhancing the accuracy of our analysis. Only articles and reviews were analyzed, excluding other publications such as conference papers and book chapters. This selection criterion was chosen to concentrate on high‐quality, peer‐reviewed sources that provide in‐depth analysis and discussion of the research topics. For the analysis, Vosviewer software was employed to create and visualize the co‐authorship network and perform keyword analysis. Vosviewer is a powerful tool for bibliometric mapping and analysis, allowing for detailed visualization of research collaboration networks and keyword co‐occurrence patterns. To ensure data consistency and quality, systematic standardization procedures were applied to author names, institutional affiliations, and country designations—a critical step in bibliometric analysis as emphasized by Selvaraj et al. (2024). Author names appearing in multiple formats across database records (e.g., “Hassan, W.” and “Hassan, Waseem”) were consolidated to a uniform nomenclature. The 274 departmental affiliations extracted from paper addresses were verified and standardized to their primary university‐level entries, eliminating redundancy from formatting variations such as abbreviations and postcode inconsistencies. Country information was standardized using ISO 3166‐1 country codes, with authors having multiple institutional locations assigned to their primary affiliation's country. This standardization improved the accuracy of institutional and country‐level collaboration patterns presented in the co‐authorship networks (Figures 3 and 4) and keyword co‐occurrence analyses. This approach provided valuable insights into the structure and focus of the research field, helping to identify critical authors, influential papers, and prevalent research themes.

## 3 Results and Discussion

### 3.1 Number of Publications

**Figure 1:**
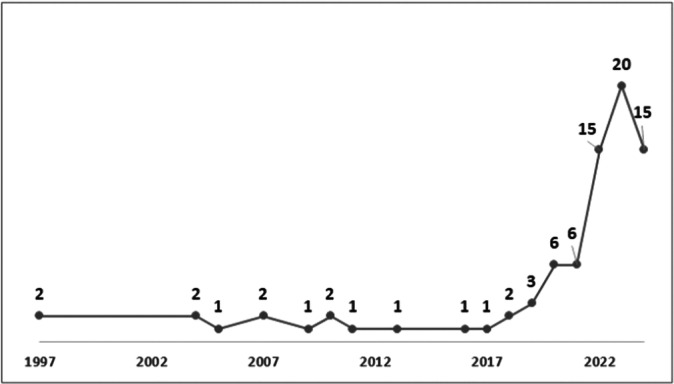
The per year number of publications per year from 1997 to July 2024.

The total number of papers published from 1997 to 2024 is 81 (Figure 1). From 1997 to 2018, the research output was sporadic and relatively low, with only a few papers published yearly. For instance, there were only two publications in 1997, one in 2005, and a couple of years with single publications scattered throughout this period. It was not until the latter half of the 2010s that a gradual increase began to be observed, with three papers published in 2019.

A significant observation is that only in 2022 did the annual publication count cross into double digits for the first time, with 15 papers published. This suggests that research in this area is relatively new and emerging. The subsequent years grew, with 20 papers published in 2023 and 15 papers in 2024 (till July 2024). This upward trend underscores the interest in exploring the applications of AI in root canal morphology, suggesting that this is a burgeoning field that will likely see further exploration and expansion shortly.

As more researchers and institutions engage in this area, it is anticipated that the body of knowledge will expand, leading to discoveries and applications that can revolutionize endodontic practices. The emerging trend indicates that AI in root canal morphology is likely to become a significant area of focus in dental research, driven by promising results and potential improvements in diagnostic accuracy, treatment planning, and patient outcomes.

### 3.2 The Authors, Universities, Journals, and Countries

In all 81 papers, 433 authors have contributed, but only two authors have published five papers each in this field: Jacobs, R. and Orhan, K. This suggests that a few key individuals are driving advancements and innovations in the integration of AI within endodontics. In bibliometric analyses, it is essential to evaluate author performance using various indicators such as the h‐index and g‐index, which provide insights into an author's impact and productivity (Hirsch 2005; Egghe 2006; Hassan et al. 2022; Hassan 2022). However, in this study, the examination of such metrics was not deemed relevant.

**Figure 2:**
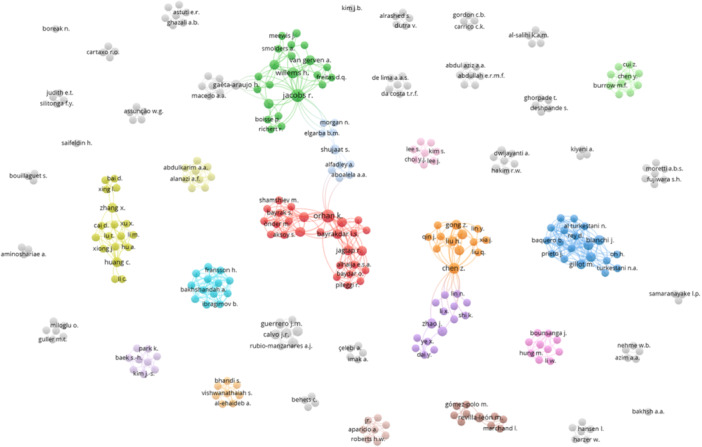
The coauthorship network for authors.

This decision was based on the finding that only a few authors have published three or five papers on AI and root canal morphology. Given the limited number of publications per author, these indicators would not provide meaningful or representative evaluations of their contributions. Consequently, a detailed analysis of the author's performance using these metrics was not conducted. Similarly, presenting collaboration networks among authors, departments, and countries is crucial for several reasons (Katz and Hicks 1997). First, it highlights the interconnectedness and collaborative efforts driving research advancements, showing how knowledge and resources are shared. Second, these networks can identify critical contributors and influential groups, providing insights into the most impactful researchers and institutions. Third, understanding collaboration patterns can reveal gaps and opportunities for fostering new partnerships, enhancing research productivity, and innovation. Finally, mapping these networks can inform funding agencies and policymakers about strategic investments and support areas, ultimately promoting a more integrated and efficient research landscape. The co‐authorship network is presented in Figure 2.

**Figure 3:**
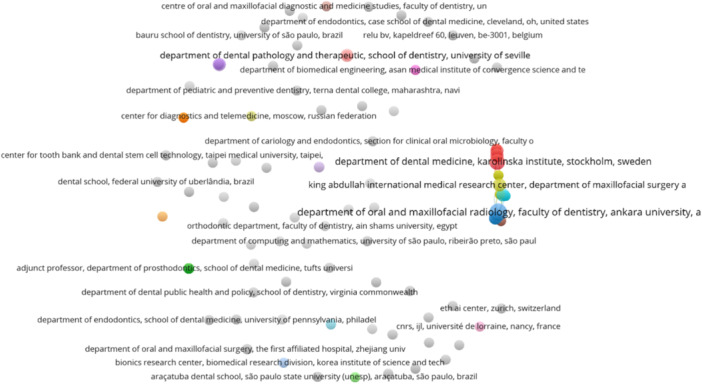
The coauthorship network for departments.

Initially, we noted 274 departmental contributions in all papers. However, several discrepancies were noted in exact addresses, i.e., the use of abbreviations, the addition of postcodes in addresses, etc. Although we presented the collaboration network among departments (Figure 3), we shifted toward university addresses. The analysis reveals a concentration of research activity among a limited number of universities. Only three universities have produced six papers in this area, i.e., KU Leuven, KU Leuven––University Hospital Leuven, and Department Beeldvorming & Pathologie. These institutions are evidently at the forefront of research in AI applications to root canal morphology, indicating a strong focus and investment in this niche area.

The 81 papers are published in 52 journals. Journal of Endodontics stands out as the primary platform for disseminating research in this field, with more than five papers published. Specifically, it has featured nine AI and root canal morphology papers. This indicates that the Journal of Endodontics is a central publication venue for researchers in this domain, providing a specialized outlet for advancements and findings.

Examining funding sources reveals that only two sponsors have funded at least three papers, i.e., the National Institutes of Health (NIH) and the National Natural Science Foundation of China. The involvement of prestigious funding agencies such as NIH and the National Natural Science Foundation of China underscores the research's high quality and significance. However, the scarcity of diverse funding sources suggests a need for more extensive financial backing from various institutions to promote a broader range of studies and encourage innovation.

Enhancing collaborative efforts, increasing funding opportunities, and encouraging contributions from diverse researchers and institutions could help address these limitations and foster a more comprehensive and inclusive body of research. The bibliometric analysis of publications addressing “Artificial Intelligence” OR “AI” AND “Root” OR “Root canal morphology” highlights a notable disparity in research contributions across different countries. Among the 31 countries identified, only three nations have made significant contributions, each publishing more than ten papers. The United States leads with 18 publications, followed by Brazil with 12 and China with 11. Together, these three countries account for a considerable portion of the total research output in this field, indicating their role in advancing the integration of AI in endodontics.

India and Saudi Arabia also exhibit some contributions, each with eight publications. Belgium and Turkey follow closely with seven publications each, while Sweden has produced six papers. Although these countries have not surpassed the ten‐paper threshold, their contributions are still noteworthy and demonstrate a growing interest and investment in AI applications in root canal morphology.

**Figure 4:**
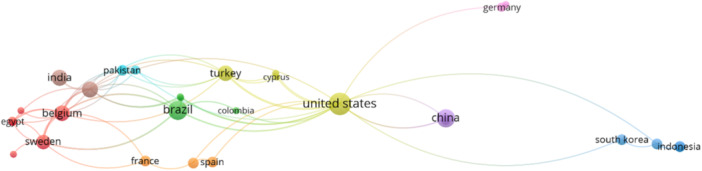
The coauthorship network for countries.

Despite the outputs from the above countries, most nations have contributed relatively few papers, with many publishing fewer than five. France, Hong Kong, Indonesia, Japan, Malaysia, Pakistan, South Korea, Spain, and Switzerland have three publications. Egypt, Germany, Iran, and the United Arab Emirates have produced two papers. The remaining countries, including Canada, Colombia, Cyprus, Denmark, Jordan, Qatar, the Russian Federation, Sri Lanka, Taiwan, Tunisia, and the United Kingdom, have each contributed only one publication to the field. The collaboration network for countries is presented in Figure 4.

The low research output from these countries raises critical questions about the barriers hindering their contributions to AI and root canal morphology research. Potential factors include limited funding for scientific research, inadequate infrastructure, and a lack of collaboration opportunities with leading institutions. Furthermore, disparities in educational resources and access to advanced technologies may also contribute to the uneven distribution of research productivity. Addressing these challenges is essential for fostering a more inclusive and globally representative body of research that can drive innovations in AI applications for endodontics. Enhancing international collaborations, increasing funding, and improving access to advanced research facilities could help mitigate these disparities and promote a more equitable distribution of scientific knowledge.

### 3.3 Co‐word Analysis of 81 Publications

Co‐word analysis is a bibliometric technique that examines the frequency and co‐occurrence of keywords in scientific literature to identify relationships and trends within a research field. It helps map a domain's conceptual structure, revealing emerging topics, research hotspots, and the evolution of scientific themes. This analysis is crucial for understanding the development of a research area and guiding future research directions (Callon et al. 1991; Ding et al. 2001; Coulter et al. 1998).

**Figure 5:**
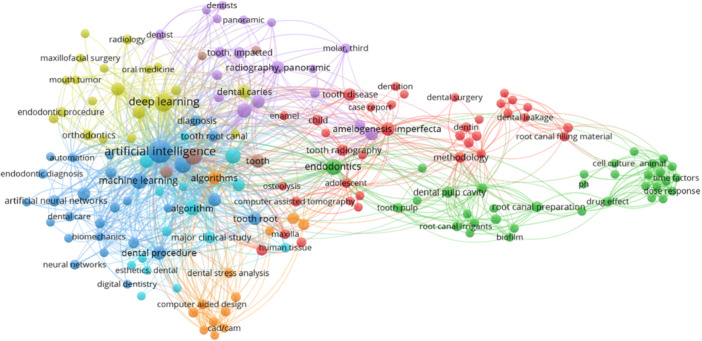
Co‐word analysis of all publications.

**Figure 6:**
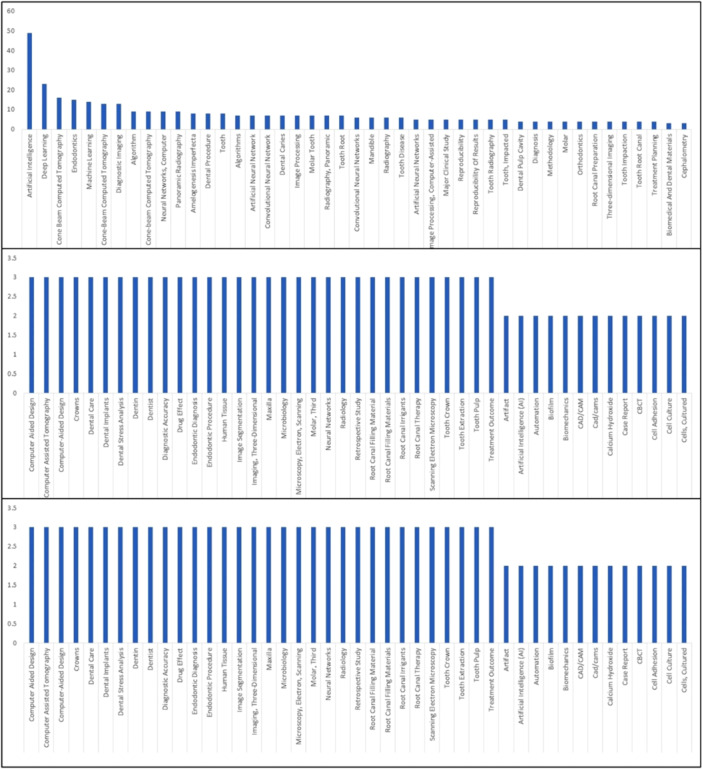
Co‐word analysis of all publications.

After taking the keywords from 81 papers (Figures 5 and 6), we performed a co‐words analysis to categorize the research focus into 25 distinct areas.

This analysis provides a detailed understanding of thematic emphasis in literature, highlighting key topics and trends. By grouping the papers into these categories, we offer a structured view of the research landscape, facilitating a comprehensive exploration of the core themes and advancements in the field. This categorization helps in elaborating the focus and trends within the collected papers.

1. Artificial Intelligence and Machine Learning
  The papers exhibit a strong emphasis on artificial intelligence (AI), with “Artificial Intelligence” appearing 49 times and “Deep Learning” cited 23 times. The frequent mentions of “Convolutional Neural Networks” (7) and “Convolutional Neural Networks” (6) suggest a focus on sophisticated machine learning models designed to analyze complex dental data. These technologies are crucial for enhancing diagnostic accuracy and developing advanced treatment algorithms in endodontics.
2. Neural Networks
  “Artificial Neural Networks” (7) and “Artificial Neural Networks” (5) reflect a significant interest in neural network models used for data analysis. The term “Neural Networks, Computer” (9) indicates a detailed exploration of these models’ applications in processing dental images and improving diagnostic processes. This focus highlights the importance of neural networks in advancing computational methods in dental research.
3. Imaging Techniques
  Imaging techniques are central to the research, with “Cone Beam Computed Tomography” (16) and “Cone‐Beam Computed Tomography” (13) being prominently featured. The inclusion of “Diagnostic Imaging” (13) and other imaging‐related terms such as “Computer‐Aided Design” (3) and “Image Processing” (7) underscores the role of advanced imaging technologies in providing detailed visualizations crucial for accurate diagnostics and treatment planning.
4. Endodontics
  Endodontics is a central focus area, with “Endodontics” appearing 15 times, highlighting its central role in the research. Keywords like “Root Canal Preparation” (4) and “Root Canal Therapy” (3) reflect detailed studies on specific endodontic procedures. The frequent mention of “Dental Pulp Cavity” (4) and “Dental Procedure” (8) further demonstrates an in‐depth exploration of endodontic practices and their clinical implications.
5. Morphology and Structure
  The category of dental morphology includes critical terms such as “Tooth Root” (7), “Tooth” (8), and “Tooth Pulp” (3). Research on “Amelogenesis Imperfecta” (8) and “Molar” (4) indicates a focus on understanding various dental structures and their developmental abnormalities. This extensive analysis of dental morphology is essential for enhancing both diagnosis and treatment of dental conditions.
6. Diagnostic Methods
  The emphasis on diagnostic methods is evident with terms like “Diagnosis” (4) and “Diagnostic Accuracy” (3). The inclusion of “Three‐dimensional Imaging” (4) and “Image Segmentation” (3) highlights advancements in diagnostic technologies aimed at improving the precision of dental assessments. These methods are crucial for accurate diagnosis and effective treatment planning.
7. Machine Learning Models
  “Machine Learning” (14) and “Deep Learning Models” (2) signify a focus on employing various machine learning techniques to advance dental research. Developing and applying these models are vital to creating innovative solutions for analyzing complex datasets and improving diagnostic and treatment procedures in endodontics.
8. Dental Materials
  Research into dental materials is represented by “Biomedical And Dental Materials” (3) and “Calcium Hydroxide” (2). The focus on “Root Canal Filling Material” (3) and “Root Canal Filling Materials” (3) highlights the study of materials used in endodontic treatments. This research is vital for selecting and optimizing materials that enhance the effectiveness and longevity of dental procedures.
9. Dental Procedures and Treatments
  Keywords such as “Dental Procedure” (8), “Dental Leakage” (2), and “Endodontic Procedure” (3) reflect a focus on various dental procedures and their outcomes. This category includes research aimed at improving procedural techniques and addressing common issues encountered during dental treatments.
10. Imaging and Analysis Technologies
  The terms “Computer‐Assisted Tomography” (3) and “Computer‐Aided Design” (3) highlight the integration of advanced imaging and design technologies. The emphasis on “Image Processing” (7) and “Image Analysis” (2) demonstrates the role of these technologies in enhancing the analysis and interpretation of dental images for better diagnostic and treatment outcomes.
11. Biofilm and Microbial Analysis
  The focus on microbial analysis is evident with terms like “Biofilm” (2) and “Colony Count, Microbial” (2). This research area explores the role of biofilms and microorganisms in dental health, particularly their impact on disease progression and treatment efficacy. Understanding microbial factors is crucial for improving dental care and prevention strategies.
12. Data Analysis and Algorithms
  The frequent mention of “Algorithm” (9) and “Algorithms” (7) underscores a focus on developing and refining computational methods for analyzing dental data. This category includes research aimed at creating algorithms that enhance data processing and interpretation accuracy and efficiency in dental research.
13. Computational Methods
  “Image Processing, Computer‐Assisted” (5) and “Image Segmentation” (3) highlight the use of computational techniques in processing and analyzing dental images. These methods are essential for extracting relevant information from complex imaging data, improving diagnostic accuracy, and supporting advanced research in endodontics.
14. Structural Analysis
  Research on structural aspects of dental components includes “Tooth Crown” (3) and “Tooth Root Canal” (4). This category focuses on analyzing the morphology and characteristics of different tooth structures, which is critical for understanding dental conditions and developing effective treatments.
15. Treatment Outcomes and Planning
  “Treatment Planning” (4) and “Treatment Outcome” (3) reflect a focus on strategies for planning and evaluating dental treatments. This research aims to improve treatment strategies and assess their effectiveness, ensuring better patient outcomes and optimized treatment protocols.
16. Radiography and Imaging
  The terms “Radiography” (6) and “Radiography, Panoramic” (7) indicate research on various radiographic techniques used in dental diagnostics. This category includes advancements in panoramic radiography and its application in evaluating dental conditions and planning treatments.
17. Clinical and Methodological Studies
  Keywords such as “Major Clinical Study” (5) and “Retrospective Study” (3) focus on different research methodologies used in clinical dental studies. This includes evaluating clinical outcomes and methodological approaches to enhance research quality and relevance in dental care.
18. Tooth and Tooth Conditions
  Research on tooth conditions includes “Tooth Disease” (6), “Tooth Extraction” (3), and “Tooth Impaction” (4). This category covers studies on various dental conditions affecting teeth and their management, highlighting the importance of addressing these issues for effective dental care.
19. Endodontic Materials
  The focus on materials used in endodontics includes “Root Canal Irrigants” (3) and “Root Canal Filling Materials” (3). Research in this category aims to evaluate and optimize materials used in root canal treatments, ensuring they meet the necessary performance and safety standards.
20. Digital Dentistry
  “Digital Dentistry” (2) reflects digital tools and technologies in dental practices, which includes research on integrating digital technologies into diagnostic and treatment processes, essential for modernizing dental care and improving efficiency.
21. Microbiology
  “Microbiology” (3) and “Enterococcus Faecalis” (2) indicate a focus on the microbial aspects of dental health. This research area explores the role of microorganisms in dental diseases and their impact on treatment outcomes, which is critical for developing effective prevention and treatment strategies.
22. Dental Prosthetics and Crowns
  Research on prosthetics and crowns includes “Dental Prosthesis Design” (2) and “Crowns” (3). This category focuses on designing and applying dental prosthetics and crowns, aiming to enhance their functionality and esthetic outcomes in dental treatments.
23. Diagnostic Imaging Techniques
  “Panoramic Radiography” (7) and “Radiography, Dental, Digital” (2) emphasize specific imaging techniques used in diagnostics. This research highlights the importance of advanced radiographic methods in providing accurate and detailed information for dental diagnosis and treatment planning.
24. Developmental and Structural Studies
  The study of craniofacial development includes “Cephalometric Analysis” (2), “Cephalometric Landmarks” (2), and “Cephalometry” (3). This research focuses on understanding craniofacial structures and their developmental changes, which are essential for various aspects of dental care and orthodontics.
25. Miscellaneous Terms
  Additional keywords such as “Case Report” (2), “Comparative Study” (2), and “Priority Journal” (2) represent various study types and classifications. These terms encompass a range of research approaches and publication types relevant to the overall understanding of AI and endodontics.

### 3.4 Limitations

This study has several limitations that should be considered when interpreting the findings. First, the analysis relied exclusively on the Scopus database, which may not capture all relevant literature. The exclusion of other databases could potentially omit pertinent studies and affect the completeness of the review.

Second, relying on Vosviewer software for network visualization and analysis introduces the possibility of errors in author names that were not manually verified. This oversight could impact the accuracy of authorship data and potentially skew the authors’ productivity and contributions assessment.

Additionally, the study focused solely on quantitative and descriptive analysis without exploring qualitative aspects or assessing the impact and quality of the research. Consequently, the analysis provides a broad overview of publication trends but lacks in‐depth insights into the significance and real‐world implications of the research findings.

To address these limitations, future studies should consider incorporating multiple databases for a more comprehensive literature review, including manual verification of author data to enhance accuracy and integrate qualitative assessments to evaluate the impact and quality of research. Such approaches will provide a more nuanced understanding of the field and contribute valuable information to the ongoing discourse on AI applications in root canal morphology.

## 4 Conclusion

This bibliometric analysis provides a comprehensive overview of the research on artificial intelligence and root canal morphology, drawing from the Scopus database. This study has elucidated the landscape of AI applications in root canal research by employing a search string that included these key terms and focusing on articles and reviews from 1997 to 2024. With a total of 81 papers published during this period, authored by 433 researchers across 31 countries, the analysis highlights the significant global interest and collaboration in this niche area of study. The 274 departmental contributions underscore the research's broad dissemination and interdisciplinary nature. Vosviewer software enabled the visualization of co‐authorship networks, revealing intricate connections among authors, institutions, and countries. This network analysis illustrates the collaborative efforts driving advancements in the field and identifies leading contributors and institutions.

Furthermore, the co‐words analysis, which categorized research into 25 distinct areas, offers valuable insights into the thematic focus and emerging trends within the literature. This detailed categorization aids in understanding the specific areas of interest and the progression of research themes over time. Overall, this study contributes to the literature by mapping the evolution of AI applications in root canal morphology research, identifying key players and trends, and providing a structured overview of the current state of knowledge in this field.

## Author Contributions

All the authors made a significant contribution to the work reported, whether that is in the conception, study design, execution, and interpretation, or all these areas; took part in drafting, revising, or critically reviewing the article; gave final approval of the version to be published; have agreed on the journal to which the article has been submitted; and agree to be accountable for all aspects of the work.

## Funding

The authors received no specific funding for this work.

## Ethics Statement

The authors have nothing to report.

## Conflicts of Interest

The authors declare no conflicts of interest.

## Data Availability

The authors have nothing to report.
